# Atypical centrioles during sexual reproduction

**DOI:** 10.3389/fcell.2015.00021

**Published:** 2015-04-01

**Authors:** Tomer Avidor-Reiss, Atul Khire, Emily L. Fishman, Kyoung H. Jo

**Affiliations:** Department of Biological Sciences, University of ToledoToledo, OH, USA

**Keywords:** centriole, centrosome, cilium, reproduction, fertilization, zygote, microtubules, sperm

## Abstract

Centrioles are conserved, self-replicating, microtubule-based, 9-fold symmetric subcellular organelles that are essential for proper cell division and function. Most cells have two centrioles and maintaining this number of centrioles is important for animal development and physiology. However, how animals gain their first two centrioles during reproduction is only partially understood. It is well established that in most animals, the centrioles are contributed to the zygote by the sperm. However, in humans and many animals, the sperm centrioles are modified in their structure and protein composition, or they appear to be missing altogether. In these animals, the origin of the first centrioles is not clear. Here, we review various hypotheses on how centrioles are gained during reproduction and describe specialized functions of the zygotic centrioles. In particular, we discuss a new and atypical centriole found in sperm and zygote, called the proximal centriole-like structure (PCL). We also discuss another type of atypical centriole, the “zombie” centriole, which is degenerated but functional. Together, the presence of centrioles, PCL, and zombie centrioles suggests a universal mechanism of centriole inheritance among animals and new causes of infertility. Since the atypical centrioles of sperm and zygote share similar functions with typical centrioles in somatic cells, they can provide unmatched insight into centriole biology.

## How to read this review and some basic definitions?

This review centers on and revolves around the formation, structure, and function of the atypical centrioles found in sexual reproduction. In particular, we discuss the hypothesis that in most animals the sperm provide the zygote with two centriolar structures; these centriolar structures may be typical or atypical centrioles. This review does not address the centriole in parthenogenetic reproduction (see for reviews Schatten, 1994; Riparbelli et al., 2010; Brevini et al., 2012). In addition, we will not discuss the critical role of the centriole in forming the sperm flagella (for revue see Chemes and Rawe, 2010; Avasthi and Marshall, 2012; Malicki and Avidor-Reiss, 2014). To help a general audience appreciate the difference between normal and atypical centrioles, the first part of the review provides a short background on centrioles and key concepts necessary to understand the similarities and differences between typical and atypical centrioles.

## Background

Centrioles and atypical centrioles can be defined using a variety of structural and functional criteria. To better understand what constitutes an atypical centriole, we start by describing the criteria that define a centriole and how the criteria have evolved from recent discoveries. We will then briefly discuss how centrioles are inherited when cells multiply by mitosis and use this as a basis for comparison to how typical and atypical centrioles are inherited during sexual reproduction.

### What are centrioles and how do we define them experimentally?

**Figure 1:**
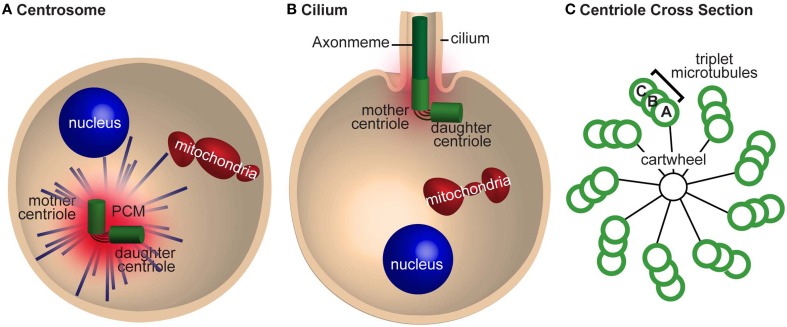
**The centrosome is required for mitosis and cilia nucleation. (A)** A cell with a centrosome emanating astral microtubules (blue lines). The mother and daughter centrioles are tethered together and are surrounded by pericentriolar material (PCM). **(B)** A cell nucleating a cilium. The mother centriole nucleates the axoneme and has less PCM and astral microtubules than in **(A)**. The centrioles are still tethered together. **(C)** A cross section of a centriole with a cartwheel and triplet microtubules with 9-fold symmetric. The microtubules in the triplet are referred to as tubules (A–C) depending on their position.

Centrioles are the subcellular structures that give rise to centrosomes, asters, and cilia. Centrioles recruit proteins from the cytoplasm that surround them in a matrix, which is known as the pericentriolar material (PCM), thus forming the centrosome (Figure 1A). The centrosome is a microtubule-organizing center that forms an aster, a radial array of microtubules that helps organize the cytoplasm. The centriole can also extend from one end to form a hair-like structure known as the cilium (aka flagellum, in sperm cells) (Figure 1B). The cilium generates cell movement and participates in cell signaling.

Our understanding and definition of a centriole changes as the technology employed to detect it improves. From the time it was identified in the late nineteenth century until the middle of the twentieth century, centrioles were identified using stains such as Heidenhain's iron haematoxylin. These initial studies found that the centrioles self-replicate, form centrosomes that emanate astral rays, and give rise to cilia.

With the advent of electron microscopy in the mid-twentieth century, centrioles were identified as barrel-shaped electron-dense structures with 9-fold radially symmetric microtubules (Figure 1C). In most cases, including the sperm and embryos of vertebrates, the centriole microtubules are organized in triplets. However, in *Drosophila*, sperm centrioles have triplet microtubules and the embryo centrioles have 9-doublet-microtubules (Figure 4 in Callaini et al., 1997); conversely, *C. elegans* sperm and embryo centrioles have 9-singlet-microtubules (Figure 3 in Pelletier et al., 2006). Altogether, regardless of the number of microtubules, most centrioles demonstrate 9-fold microtubule symmetry.

Electron microscopy better defined the structural role of the centriole in the centrosome, cilia, and during self-replication. In cilia, the centriolar microtubules elongate to form the axoneme—the backbone of the cilium (Figure 1B). The axoneme is also made of 9-fold symmetric microtubules, but unlike most centrioles, they are made of doublet microtubules. In the centrosome, the centriole is surrounded by the PCM, which emanates astral microtubules (Figure 1A). During self-replication of centrioles (centriole duplication), a new centriole bud forms perpendicular to the proximal end of the preexisting centriole; the centriole bud is known as the procentriole. This procentriole has a core cartwheel (Figure 1C). In animals, the cartwheel is either lost in mature centrioles or restricted to the proximal end. Altogether, these electron microscopy studies gave rise to the notion that 9-fold symmetry of centrioles is important for the organization of the cilium but did not define a role for 9-fold symmetry in centrosome formation or self-replication. In addition, it is apparent that the cartwheel is a transient scaffold structure that mediates the first step in centriole formation (Nakazawa et al., 2007; Guichard et al., 2010; van Breugel et al., 2011).

In the late twentieth century, antibodies against PCM and microtubular proteins (i.e., tubulin) were used to observe centrosomes (Heidemann and Kirschner, 1975; McGill and Brinkley, 1975; Connolly and Kalnins, 1978). Using these antibodies alone, in the absence of electron microscopy, the centriole cannot be identified directly; however, the presence of centrioles can be inferred from the ability to form PCM and to nucleate astral microtubules (Sluder, 2014). These inferences must be treated with caution, as tubulin and centrosome-enriched proteins can form centrosome-like structures lacking centrioles. These structures are named acentriolar centrosomes and were reported in the spindle poles of female meiosis (Schatten et al., 1985; Calarco, 2000). Therefore, the use of antibodies against centrosomal and microtubular proteins is insufficient to identify a centriole, and definitive identification of centrioles requires complementary electron microscopy studies.

In the beginning of the twenty-first century, centriole-specific proteins were identified, such as: Sas-4, Sas-6, Cep135/Bld10, Ana1/Cep295, Ana2/Sas-5/Stil (Kirkham et al., 2003; Dammermann et al., 2004; Matsuura et al., 2004; Goshima et al., 2007) (**Figure 3A**). These advances allowed for the identification of centrioles, either by using antibodies against these proteins or by genetically adding fluorescent tags. A centriole appears either as a focus of centriolar proteins surrounded by PCM proteins, which emanates astral microtubules, or as a focus of centriolar proteins at the base of the cilium. However, overexpression of some of the genetically tagged centriolar proteins can produce artificial centrosome-like structures (Rodrigues-Martins et al., 2007a; Gopalakrishnan et al., 2011). Therefore, definitive identification of centrioles still requires complementary electron microscopy studies when centriolar proteins are overexpressed.

Finally, in the last few years, the development of Super-Resolution light microscopy has allowed for detailed visualization of centriolar protein organization at previously unachievable resolution (Fu and Glover, 2012; Lau et al., 2012; Mennella et al., 2012). With the aid of this technology, for example, it has been determined that the PCM is not as amorphous as it was previously thought, and that it can be distinguished from shells that surround the centriole. The first shell, the PCM tube, contains Asterless/Cep152, and the outer PCM contains γ-tubulin (Mennella et al., 2012) (**Figure 3C**). Furthermore, it was found that some centrosomal proteins also exhibit semi-9-fold symmetry (Lau et al., 2012; Mennella et al., 2012). In time, this technology should allow us to define a centriole based on relative localization of centriolar proteins.

In summary, currently there is only one generally-accepted, definitive criterion for the identification of centrioles: a centriole is a subcellular structure characterized by a barrel shape and made of 9-fold symmetric microtubules that is visible using electron microscopy. However, a centriole can be inferred experimentally via alternative techniques using five criteria: (1) the centriole duplicates to form one centriole per cell cycle; (2) the centriole recruits PCM; (3) the centriole is surrounded by astral microtubules; (4) the centriole elongates to form a cilium; and (5) the centriole is made of centriole-specific proteins with a particular organization. As we discuss below, many of the centrioles during reproduction are atypical and are not made of 9-fold symmetric microtubules; yet, they do meet most of the other criteria and can therefore be considered centriolar structures.

### How does the centriole function in cells and how is it maintained when cell divides?

In this section, we will address how centrioles, centrosomes, asters, and cilia form and function in a typical cell. At the end of this section we will briefly point out the differences between typical somatic centrioles and those atypical one formed during reproduction.

**Figure 2:**
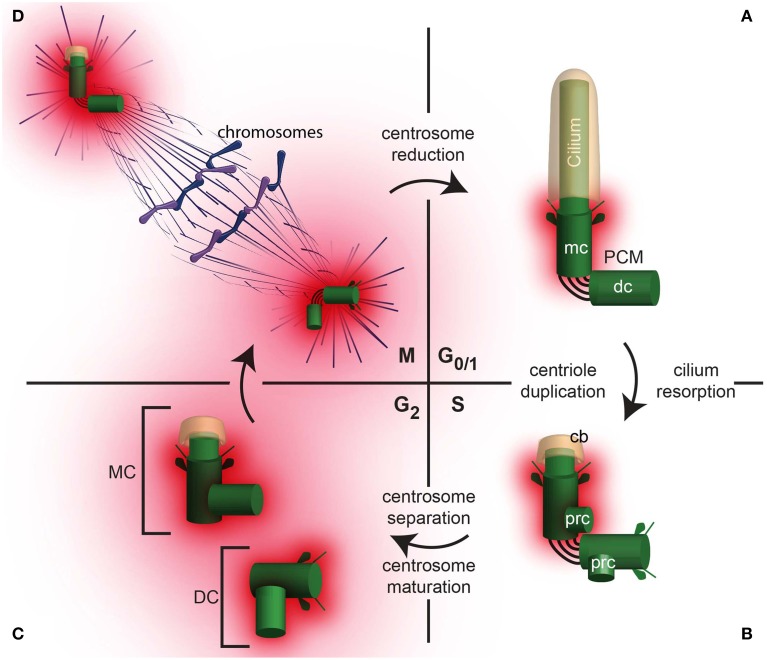
**The centrosome changes throughout the cell cycle. (A)** A daughter cell inherits one centrosome, which then undergoes centrosome reduction, where the PCM quantity is reduced and the centrosome migrates to the cell periphery where it nucleate a cilium in G_0/1_. In G_0/1_, the centrioles (mc, mother centriole; dc, daughter centriole) are tethered together, with PCM surrounding the mother centriole. **(B)** In S phase, the cilium is reabsorbed, leaving a ciliary bud (cb). Coincident with DNA replication in the nucleus, procentrioles (prc) form, each one orthogonal to the mother or daughter centriole, respectively. **(C)** In G_2_, the tether between the mother and daughter centriole is severed, allowing the centrosomes to separate. The centrosome containing the original mother centriole is often called the Mother Centrosome (MC), whereas the original daughter centriole is now part of the Daughter Centrosome (DC). Both the Mother Centrosome and Daughter Centrosome recruit more PCM. **(D)** In M phase, the centrosomes migrate to opposite poles of the cell and regulate chromosome segregation.

Cells have two centrioles during early interphase and four centrioles after S phase. These centrioles are not identical and have distinct structure and functions during the cell cycle (Figure 2). Their various functions include cilium formation, centrosome assembly, and self-replication. In non-dividing somatic cells, in the G_0_/G_1_ phase of the cell cycle, the older centriole in the cell (the mother centriole) is docked to the cell plasma membrane and forms a cilium, a cellular extension that has motile and sensory functions and is in a separate compartment from the cytoplasm (Figure 2A). In these cells, the mother centriole is also known as a basal body; and the cilium, if it is motile, as it is in the sperm cell, is sometimes referred to as a flagellum. The daughter centriole is tethered to the mother centriole; its function, if any, in G_0_/G_1_ is not clear. However, the daughter centriole's main role is to develop along the cell cycle into a fully functional centriole that will be the mother centriole of one of the daughter cells following the next mitosis. Therefore, having the daughter centriole in differentiated cells is important for centriole formation and inheritance during cell division.

Once a cell obtains the signal to prepare for cell division, the two centrioles start to modify dramatically (Figure 2B). First, the cilium is partially or completely resorbed, allowing the two centrioles to be internalized. Then, during the S phase of the cell cycle, each of the centrioles duplicates. The result of this duplication is two centrosomes, each containing a pair of centrioles. The two centrosomes are loosely attached to each other via links (interconnecting fibers). Each centriole pair contains mother centriole and a daughter centriole; the daughter centriole is attached to the wall of the older centriole.

During the G2 phase, following centriole duplication in S phase, the two pairs of centrioles separate and form independent centrosomes (Figure 2C). Sometimes, the centrosome that contains the original mother centriole is referred to as the mother centrosome, and the other, which contains the original daughter centriole, is referred to as the daughter centrosome. In each centrosome, the older centriole recruits additional PCM in a process that is referred to as centrosome maturation (Palazzo et al., 2000). During maturation, the centrosome increases its capacity to nucleate and anchor microtubules. These microtubules, known as astral microtubules, emanate from the centrosome in an astral pattern.

During cell division, (M phase), the two centrosomes interact with the cell division apparatus and become part of the mitotic spindle poles via some of their astral microtubules. The remaining astral microtubules connect the spindle pole to the cell plasma membrane and orient the axes of cell division (reviewed in Stevermann and Liakopoulos, 2012). During anaphase stage of the M phase, each pair's centrioles lose their perpendicular orientation (i.e., become disengaged). However, the centrioles are still connected via interconnecting fibers, which keep them near each other (Bahe et al., 2005; Yang et al., 2006). At the end of cell division, the astral microtubules and PCM are partially diminished in a process that is referred to as “centrosome reduction” (Manandhar et al., 2005). Once the cell division is complete, each of the two daughter cells has a centrosome with a pair of centrioles, which in the next G1, migrates to the cell plasma membrane to form a cilium.

Sexual reproduction consists of many exceptions to the precedential expectations of normal cell cycle; these exceptions may explain the presence of atypical centrioles that arise during gametogenesis and fertilization. This deviation starts with meiosis, continues with gamete differentiation and fertilization, and ends at the finish of zygotic cell division. For example, the centrioles duplicate during meiosis or early in spermiogenesis independently of DNA duplication, and the centriole can stay connected to the cilium while mediating meiotic and zygotic cell division (Riparbelli and Callaini, 2010; Riparbelli et al., 2012; Gottardo et al., 2013). Also, in sperm cells, the centriole is attached to the nucleus, and the proximal part of cilium is open to the cytoplasm (Basiri et al., 2014). These and other differences from mitotic cell division may explain the presence of atypical centrioles during reproduction.

### Why do centrioles duplicate?

In most cells, a new centriole forms once per cell cycle near a preexisting centriole; this is referred to as centriole duplication. Centriole duplication provides a way to assure that cells have precisely two centrioles (Delattre and Gonczy, 2004). However, in some cells and in some circumstances, centrioles form in the absence of a preexisting centriole, a process which is referred to as *de novo* centriole formation (La Terra et al., 2005; Rodrigues-Martins et al., 2007b). It has been shown that when centrioles form *de novo*, the number of centrioles is more than two. Having too many centrioles is a devastating condition to animal development (Holland et al., 2010; Godinho et al., 2014). For example, having too many centrioles results in having too many cilia, which interferes with the cell's sensory function (Mahjoub and Stearns, 2012). Having precisely two centrioles is also important for accurate cell division. Having only a single centriole results in monopolar spindles and mitotic arrest, (Kirkham et al., 2003) and having more than two centrioles results in multipolar spindles and damage to chromosomes (Ganem et al., 2009). Thus, in many cases, cells need to control their centriole number precisely.

Utilizing a centriole duplication mechanism in which each preexisting centriole gives rise to only one new centriole per cell cycle can control centriole number. The molecular mechanisms that ensure that only one new centriole forms per preexisting centriole is another intensively researched subject (for recent review see Sluder and Khodjakov, 2010; Fong et al., 2014). Since centriole number is so precisely controlled during development, and the zygote gives rise to all other cells of an animal, it is expected that during reproduction, centrioles will form via centriole duplication. In the absence of typical centrioles, centriole duplication in some organisms is mediated by atypical centrioles that, although structurally different from centrioles, act as the preexisting centriole for the purpose of promoting assembly of a new “typical” centriole (Blachon et al., 2014; Lee et al., 2014).

**Figure 3:**
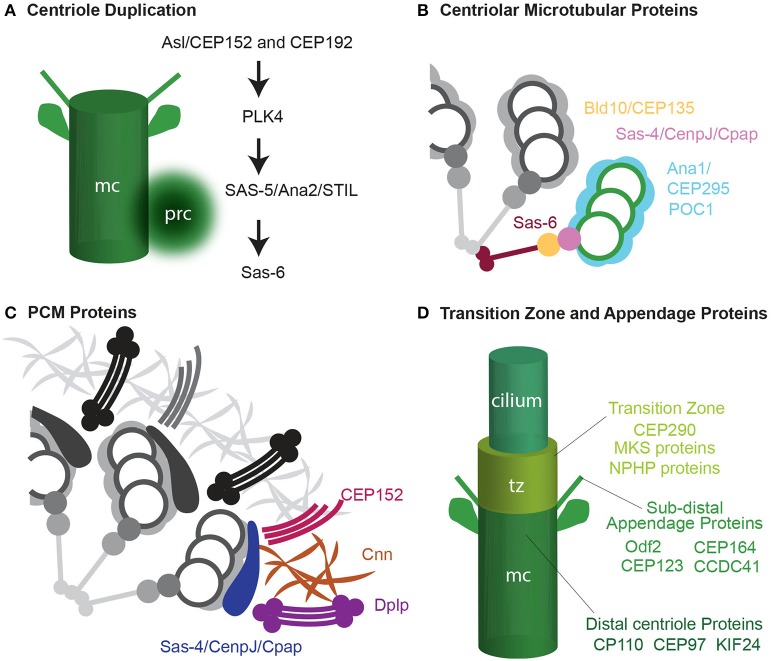
**Four groups of centrosomal proteins. (A)** The cascade of proteins required for initiating the formation of the procentriole (prc) near the proximal end of the mother centriole (mc). **(B)** A quarter of a cross-section of a centriole, showing cartwheel and microtubule wall proteins. **(C)** A quarter of a cross-section of a centriole, showing a matrix of PCM proteins. Adapted from Mennella et al. (2012). **(D)** Transition zone (tz), subdistal appendages, and distal centriole proteins that are required for cilium formation.

How centrioles form and the molecular mechanisms used during their formation are intensely investigated subjects that have been reviewed recently (Nigg and Stearns, 2011; Gonczy, 2012; Avidor-Reiss and Gopalakrishnan, 2013; Jana et al., 2014; Winey and O'Toole, 2014). In general, there are four groups of centrosomal proteins based on their function in centriole assembly and function. One that mediates the initiation of centriole biogenesis by cartwheel formation (Figure 3A); another that functions in centriole microtubule formation, stabilization, and elongation and forms a centriole processor known as the procentriole (Figure 3B); one that is responsible for recruiting and anchoring PCM and transforms the daughter centriole to an independent centrosome (Figure 3C); and finally, one that is responsible for cilium formation (Figure 3D). As described below, the formation of the atypical centrioles involved with fertilization (e.g., spermatozoa centriole, the PCL, and the degenerated centriole) begins, in all cases, with the same centriolar proteins described above. However, their formation does not continue along the canonical process of centriole; instead, assembly is arrested as a centriole precursor. Alternatively, the centrioles, after forming to completion, may add an additional stage where they are dramatically modified (see below: centrosome reduction).

## Centriole inheritance during reproduction

Centrioles, centrosomes, asters, and cilia were first discovered in the context of reproductive biology (Wheatley, 1982; Scheer, 2014; Sluder, 2014). Yet, despite a century-long realization that centrioles are fundamental to the initiation of new animal life, the mechanism of centriole inheritance during fertilization and the precise composition of zygotic centrioles remain unclear. Here, we will describe the fate of the gamete centrioles and their function after fertilization. We will discuss the idea that zygote centrioles are a group of subcellular structures that share a common mechanism of formation, yet can deviate during their development to have distinct structures and protein compositions. These unique properties may serve a role in the specialization of the centriole for zygotic functions. However, ultimately all centriolar structures, typical and atypical, share a common function: to replicate and form one, and only one, centriolar structure per cell cycle. Therefore, the presence of atypical centrioles provides the framework for a universal mechanism of centriole inheritance among animals.

For the purpose of this review, the term “reproduction” includes gametogenesis, fertilization, and zygote development; “embryo development” starts post-fertilization and includes zygote development and later developmental stages. Because “centriole” and “basal body,” as well as “cilium,” “flagellum,” and “the sperm tail” are related terms referring to the same structures, we will simply use the terms “centriole” and “cilium,” respectively. Since here we discuss various types of centrioles, we will use the term “centriole” to refer to the typical centriole with 9-fold symmetry; we will refer to centriole-related structures that deviate from that definition as “atypical centrioles”; and we will use the term “centriolar structures” to refer collectively to both centrioles and atypical centrioles.

### How do the embryos inherit their centrioles?

**Figure 4:**
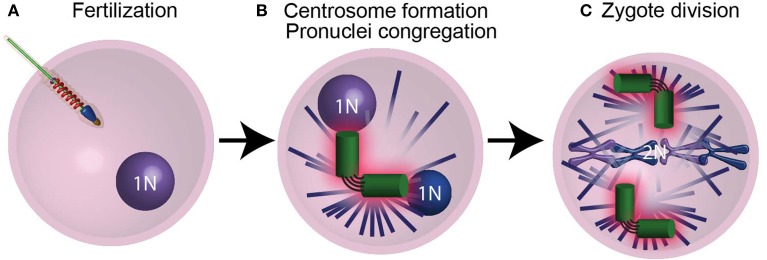
**The centrioles are essential for pronucleus migration. (A)** After the sperm fertilizes the ovum, it provides the zygote with modified centrioles and its genetic material in the form of a male pronucleus. **(B)** The centrioles (green) then recruit maternal PCM proteins (pink) and nucleate astral microtubules (purple lines), which mediate the migration of pronuclei (blue spheres) using motor proteins. **(C)** Following duplication, the centrosomes found in the spindle poles mediate chromosome segregation. N and 2N indicate chromosome copy number.

The first cell of an animal embryo (the zygote) forms not by mitosis as most cells do, but by the fusion of male and female gametes (ovum and spermatozoa) in the process of fertilization (Figure 4A). This difference in the mechanism of cell formation raises the question: how does the zygote obtain its centrioles? It is well established that in most animals no functional centrioles capable of duplication are present in mature female gametes, and in many animals centrioles are eliminated or inactivated during ovum formation (oogenesis) (reviewed by Manandhar et al., 2005; Sun and Schatten, 2007; Mikeladze-Dvali et al., 2012). It is also believed that in most animals, mature male gametes have some centrioles or centriole-derived structures (Sutovsky and Schatten, 2000; Schatten and Sun, 2011). These paternal centrioles form a centrosome in the zygote that is essential for zygote function and embryo development (Sathananthan et al., 1996; O'Connell et al., 2001; Stevens et al., 2007; Varmark et al., 2007). In addition, the zygote is the first cell of an animal, and it produces all other cells in an animal by dividing mitotically. When the zygote prepares to divide, the zygote centrioles duplicate as they do in normal somatic cells (Callaini and Riparbelli, 1996; Sathananthan et al., 1996; O'Connell et al., 2001). Therefore, the sperm centrioles, which become the zygotic centrioles, are the origin of virtually all of the centrioles in an animal.

### What is the role of the zygotic centrioles?

The zygote centrosome forms when the paternal centrioles recruit maternal PCM protein (Figure 4B) (Sluder and Rieder, 1985b; Stearns and Kirschner, 1994; Callaini and Riparbelli, 1996; Terada et al., 2010; Schatten and Sun, 2011). This centrosome acts as a microtubule-organizing center that assembles a large microtubule aster. The zygote centrosome is thought to have two important functions: first, it mediates the migration and congregation of the female and male pronuclei (Figure 4B); second, it assists in zygote division (Figure 4C).

The role of the zygotic centrosome in pronuclei migration and congregation is a specialized function unique to the zygote, and it appears to be conserved in many animals (Schatten et al., 1986; Riparbelli et al., 2000; Sutovsky and Schatten, 2000). Pronucleus migration happens in one of two ways: the first, in which the male pronucleus migrates; and the second, in which the female pronucleus migrates. *C. elegans* falls into the former group, where the centrosome mediates male pronucleus migration toward the female pronucleus (Kimura and Onami, 2005). Sea urchin, cattle, and *Drosophilae* fall into the latter, where the centrosome mediates female pronucleus migration toward the male pronucleus (Navara et al., 1994; Fechter et al., 1996; Riparbelli et al., 2000).

The role of the centrosome during zygote cell division may also be specialized. In somatic cells, the centrosome increases the fidelity of cell division, but cell division can take place in the absence of centrosomes (Debec and Abbadie, 1989; Hinchcliffe et al., 2001; Khodjakov and Rieder, 2001; Basto et al., 2006) #3906. However, there is evidence which suggests that unlike in somatic cells, centrosomes are essential for cell division in the zygote and in early embryonic cells in *Drosophila melanogaster*, *C. elegans*, and sea urchin (Sluder et al., 1989; O'Connell et al., 2001; Stevens et al., 2007; Varmark et al., 2007). Such an essential role of the centriole needs to be shown in human and other mammalian zygotes. This precaution is necessary, as mouse embryos are thought to have no centrioles up to the 32-cell stage (Schatten et al., 1986; Gueth-Hallonet et al., 1993), and previous studies in humans and other mammals showed that acentriolar zygote development in parthenogenetic embryos can occur to some extent, but eventually these embryos fail and die (Paffoni et al., 2007; de Fried et al., 2008).

### What happens to the centriole upon entry into the zygote?

As discussed below the sperm provide either one of two centrioles that are known as the distal centriole and proximal centriole. Immediately after fertilization the centriole may or may not be released from the sperm to form the zygotic centrosome. In sea urchin, the distal centriole is detached from the flagellum's axoneme (Fechter et al., 1996), but remains firmly attached to a depression in the nuclear envelope of the male pronucleus (i.e., centrosomal fossa) (Paweletz et al., 1987). However, detachment from the axoneme does not occur in all animals, and the zygotic centriole of *D. melanogaster* stays connected to the sperm flagellum (Riparbelli and Callaini, 2010). Maintaining the connection to the axoneme or centrosomal fossa inhibits the zygotic centrosome movement toward the female pronucleus, and instead the female pronuculeus must migrate to allow pronuclei congression. Whether or not the distal centriole detached in humans is not clear (Simerly et al., 1995). However, the human's proximal centriole is released by 26S proteasome from the connecting piece in which it is embedded (Rawe et al., 2008). Whether the proximal centriole is released from its centriolar adjunct that resembles a simple axoneme is not clear.

Fertilization of the ovum by the sperm occurs during female meiosis. Since both the ovum and the sperm have microtubular networks, a mechanism to prevent interference between the two-microtubule systems may be necessary. One such mechanism is that the recruitment of the maternal PCM proteins to the sperm centrioles takes place only after completion of female meiosis in *C. elegans* (McNally et al., 2012). This type of regulation may not take place in Drosophila where centrioles with PCM and aster are observed during meiosis (Callaini and Riparbelli, 1996; Riparbelli et al., 1997; Blachon et al., 2014); however, this was not studied in detail and more study directed to address that and the role of atypical centrioles in this regulation are needed.

### When does the ovum eliminate or inactivate its centrioles, and why does it do so?

**Figure 5:**
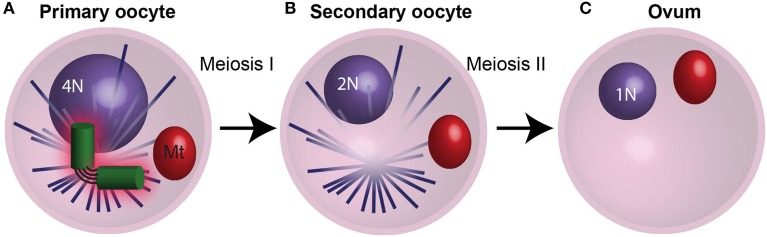
**The centrosome is eliminated during oogenesis. (A)** During meiosis I in humans, the 4N primary oocyte divides and the centrioles degrade during prophase I. **(B)** The resulting secondary oocyte then undergoes meiosis II in the absents centrioles. **(C)** The culmination of oogenesis is an ovum with an unduplicated genome (1N), which lacks centrioles. Mt, mitochondria. N and 2N indicate chromosome copy number.

In most animals, it is thought that the zygote centrioles that replicate and develop into embryo centrioles are derived from paternal centrioles. For this to happen, the maternal centrioles are lost or inactivated, so they are unable to participate in meiotic spindle assembly during oogenesis or duplicate in the zygote (Schatten, 1994). Oogenesis starts when female primordial germ cells undergo mitosis and differentiation to form an oogonium, then oocyte, and finally the ovum (Figure 5). The oogonium proliferates by mitosis and forms primary oocytes. Then, each oocyte undergoes Meiosis I and II to form the ovum—the mature egg. During mammalian oogenesis, centrioles are present up to mid-Meiosis I (the pachytene stage), but are absent in the meiotic spindles of oocytes (Figure 5A) (Szollosi et al., 1972; Sathananthan et al., 2006; Luksza et al., 2013). In contrast, in echinoderms (e.g., starfish and sea urchin), centrioles are present in female meiosis, and they are eliminated during polar body formation or lose their ability to duplicate (Sluder and Rieder, 1985a; Sluder et al., 1989; Nakashima and Kato, 2001; Uetake et al., 2002; Shirato et al., 2006) (Figures 5B,C). Altogether, regardless of whether maternal centrioles are present or absent during female meiosis, they neither function in zygote mitosis nor do they contribute to the embryo. During parthenogenesis, on the other hand, the zygote centrioles are of maternal contribution (Washitani-Nemoto et al., 1994). Since retention of maternal centrioles leads to parthenogenesis, it is thought that elimination of the maternal centriole is a mechanism to assure that parthenogenetic development does not take place, (Washitani-Nemoto et al., 1994; Manandhar et al., 2005). Furthermore, failure to eliminate maternal centrioles has been reported to result in multipolar mitotic spindles in the *C. elegans* zygote; therefore maternal centriole loss or inactivation is essential for normal embryo development (Kim and Roy, 2006).

### How do sperm cells obtain their centrioles?

**Figure 6:**
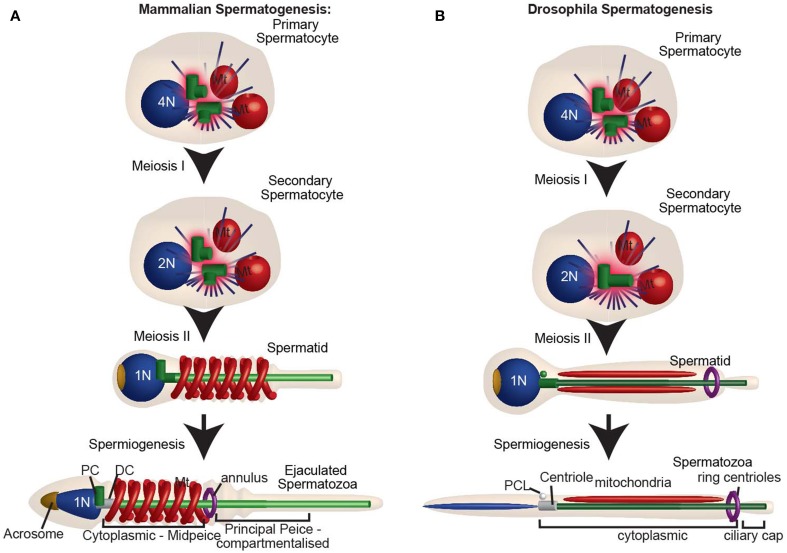
**The centrosome is reduced during spermatogenesis. (A)** Mammalian spermatogenesis: In the primary spermatocyte, the two centrioles duplicate as the DNA replicates. After meiosis I, the secondary spermatocyte with two centrioles duplicates, while the DNA does not. For this reason, Meiosis II culminates with two centrioles in each of the spermatids. The spermatid then undergoes spermiogenesis, during which the distal centriole forms a flagellum, and the centrosome is reduced. Human ejaculated spermatozoa have one intact centriole and one degenerated centriole. **(B)** *Drosophila* spermatogenesis: Spermatogenesis in flies. Note that during meiosis I and meiosis II the centrioles do not duplicate, and each spermatid has only one centriole (c). This centriole then buds off the PCL and gives rise to a flagellum. Both the centriole and PCL loses most of their component proteins, as part of centrosome reduction. PC, proximal centriole; DC, distal centriole; Mt, mitochondria. N and 2N indicate chromosome copy number.

Spermatozoa are formed through a long differentiation process called spermatogenesis. Spermatogenesis begins with the differentiation of a stem cell into a spermatogonium, then a spermatocyte, spermatid, and finally spermatozoa (Figure 6). All of these sperm cell types, with the exception of spermatids and spermatozoa, are produced by mitotic division with typical centriole duplication and have two centrioles when resting and four centrioles when preparing for cell division (Figure 6A). In contrast, spermatids are formed as a result of two rounds of specialized cell divisions, meiosis-I and meiosis-II. In spermatids, the numbers of centrioles vary from animal to animal.

During spermiogenesis, the centrioles are located beneath the nucleus in the neck region of the spermatid. The two centrioles of vertebrate spermatids have distinctive functions and positions and, therefore, have specific names based on these aspects: the proximal centriole and the distal centriole (Figure 6). Confusingly, there are two naming systems assigned to these centrioles; the one used only in the sea urchin (Longo and Anderson, 1969), and the most common one used in other vertebrates (Longo and Anderson, 1968; Marshall and Luykx, 1973). Here we will use the common nomenclature, in which the distal centriole is the centriole that templates the sperm flagellum. The proximal centriole either forms a short cilium-like structure called the centriole adjunct or remains static (Figure 6 in Fawcett and Phillips, 1969). The proximal centriole is the daughter of the distal centriole, and in non-insect animals, it is thought to be formed between the two meioses (Figure 1 in Rattner, 1972; Krioutchkova and Onishchenko, 1999; Peters et al., 2010). This centriole duplication is unique in that it takes place in the absence of DNA duplication.

In insects, it was thought for a long time that spermatids possess only one centriole (Phillips, 1970; Szollosi et al., 1972; Szollosi, 1975; Tokuyasu, 1975; Friedlander, 1980). This centriole is homologs to the vertebrate distal centriole that templates the sperm flagellum. Since this centriole is longer than a typical centriole in some species, it is sometimes referred to as the giant centriole. Recently, it was discovered that in addition to the giant centriole, *D. melanogaster* sperm have a unique atypical centriolar structure named the proximal centriolar-like (PCL) (Figure 6B) (Blachon et al., 2009, 2014; Mottier-Pavie and Megraw, 2009; Stevens et al., 2010). The PCL appears early in spermiogenesis and therefore, like the vertebrate proximal centriole, its formation takes place in the absence of DNA formation. However, whether or not the PCL is found in other animal species is unknown.

### What is centrosome reduction?

During the differentiation of the spermatid cells into spermatozoa (spermiogenesis), the centrosome loses many of its characteristics in a process known as centrosome reduction (Manandhar et al., 2005) (**Figure 8**). Centrosome reduction is a conserved process found in all animals studied. However, the extent to which the centrosome reduces, and the details of the process varies between species. It ranges from merely loosing the PCM, as in *C. elegans*, to intermediate situation, where the centrioles lose their 9-fold symmetry and, to an extreme case, where the two sperm centrioles are entirely degenerated, as in mice. Centrosome reduction is a continuous process, however, a few benchmark steps have been distinguished. First, the spermatid centrosome loses its capacity to nucleate astral microtubules. Then, the centrosome loses its PCM and some centrioles lose many of their protein components. Finally, some centrioles undergo structural modification, resulting in a loss of their 9-fold-symmetry and microtubules. These centrioles are thought by many to be degenerated and non-functional (Manandhar et al., 2000, 2005; Delattre and Gonczy, 2004).

Centrosome reduction occurs in a variety of species, including mammals, mollusks, and insects. However, centrosome reduction was only studied systematically in rhesus monkeys (Manandhar and Schatten, 2000), mice (Manandhar et al., 1998), and *Drosophila* (Blachon et al., 2014). Centrosome reduction was observed by three methods: electron microscopy, immuno-labeling of centrosomal components, and genetic tagging of centrosomal proteins. Immuno-labeling of the PCM proteins (γ-tubulin, and pericentrin) failed to detect any proteins in the mouse and rhesus monkey spermatids. Electron microscopy identified structural changes that ranged from apparent elimination of both centrioles (Woolley and Fawcett, 1973) to partial structural defects in the distal centriole, but with an intact proximal centriole (Zamboni and Stefanini, 1971) (**Figure 8C**). This partial structural defect in human distal centriole results in the absence of 50% of the centriolar microtubules (Manandhar and Schatten, 2000). Likewise, the amount of the centriolar protein centrin is reduced in the rhesus distal centriole, but not in the rhesus proximal centriole (Manandhar and Schatten, 2000). In *D. melanogaster* spermatozoa, PCM proteins γ-tubulin and Cnn, as well as centriolar proteins Ana1, bld10, Sas-4, Sas-6, and Ana2, are diminished (Wilson et al., 1997; Li et al., 1998; Blachon et al., 2014) (Figures 8D,E). However, mass spectroscopy analysis of *D. melanogaster* spermatozoa identifies several core centriolar proteins (e.g., Ana1, Ana3, and Bld10) (Wasbrough et al., 2010). This suggests that small amounts of centriolar proteins are present in the spermatozoa and may function in the zygote.

The presence of centrosome reduction in all studied animals suggests that centrosome reduction is an essential and active process. One prevailing hypothesis is that the role of centrosome reduction is to inactivate the centrosome such that it will require activation once entering the centrosome-deficient oocyte (Riparbelli et al., 2010; Schatten and Sun, 2011; Mikeladze-Dvali et al., 2012). This activation is accomplished by the sperm centriole recruiting oocyte PCM proteins (Pelletier et al., 2004; Dix and Raff, 2007). However, it is unknown if centrosome reduction is essential for male fertility, or if it contributes to human diseases.

The loss of the centriolar microtubules, or centriolar 9-fold symmetry, in spermatozoa centrioles during centrosome reduction in animals can be explained in two distinct ways. In one explanation 9-fold symmetric microtubules are not important in the zygote, and there was no evolutionary pressure to keep centriole symmetry; therefore, this property was lost. In the second, the absence of 9-fold symmetry has evolutionary advantage. One possible reason to maintain 9-fold symmetry microtubules in spermatozoa centrioles is that it is essential in the spermatozoa in the sperm neck region. For example in non-rodent mammals the proximal centriole may be essential for organizing the sperm connecting piece, a specialized form of PCM. The proximal centriole may also be essential for the centriolar adjunct, a specialized form of axoneme. Another possible reason for maintaining the 9-fold symmetry is that it is essential to form a functional cilium early in embryogenesis (Hiraki et al., 2007). In mice that have no centrioles in spermatozoa, centrioles appear in embryos at the 32-cell stage and cilia appear shortly after at the 64-cell stage (Bangs et al., 2015). In vertebrates that are thought to have at least one centriole in the spermatozoa, cilia play an essential role in embryogenesis, but when cilia appear in embryogenesis is not known (Goetz and Anderson, 2010). On the other hand, *Drosophila*, which appears to have atypical centrioles in spermatozoa, does not have cilia during early embryogenesis (Avidor-Reiss et al., 2012). Therefore it is possible that 9-fold symmetric microtubules in spermatozoa are maintained to allow for cilia formation early in embryogenesis and was lost in animals that do not have cilia in early embryogenesis.

In all of the studied model organisms, the molecular mechanisms of centrosome reduction remain unknown. Several mechanisms may mediate centrosome reduction: (1) depletion of centrosomal components due to halting of transcription and/or translation; (2) centrosomal protein degradation; (3) ectocytosis during spermiation, when most of the sperm cell's cytoplasm is remodeled and discarded in the waste bag (O'Donnell et al., 2011); (4) autophagy of centriole components (Pampliega et al., 2013; Tang et al., 2013); (5) post-translational modification that destabilizes the ability of centrosomal proteins to bind to the centrosome. These mechanisms are not mutually exclusive and may work together in some combination to affect various components of the centrosome.

### How many centrioles does the sperm provide to the zygote?

**Figure 7:**
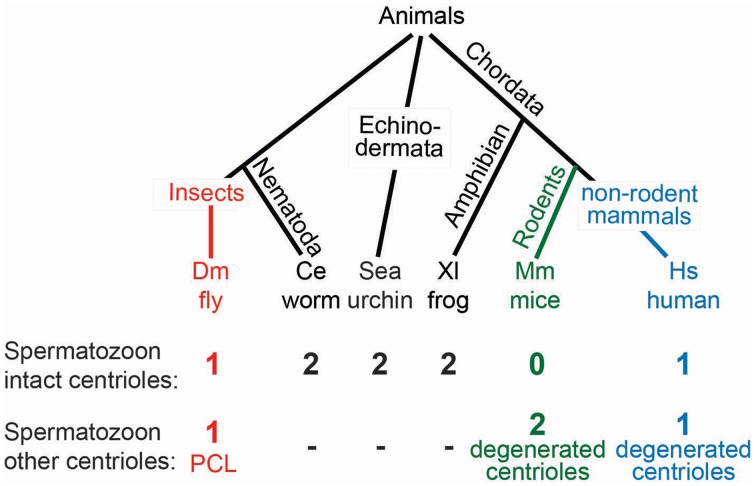
**Models for centriole number in sperm vary from organism to organism**. A phylogenetic tree with the varied centriole numbers in different organisms. Organisms with two intact centrioles in the spermatozoa (black). Organisms with one centriole and one PCL (red). Organisms with no centrioles in spermatozoa (green). Organisms with one centriole and one degenerated centriole in spermatozoa (blue).

Since all somatic centrioles originate from the zygotic centrioles, it is not surprising that the zygote also possesses two centrioles. Likewise, because the zygotic centrioles originate from the spermatozoa, it is unsurprising that the spermatozoa of many animals appear to have two centrioles (Figure 7) (Krioutchkova and Onishchenko, 1999). Indeed, it was proposed that the ancestral sperm cells of animals had two centrioles with 9-fold symmetric microtubules (Baccetti and Afzelius, 1976). However, in several animal groups, including humans, mammals, and insects, exceptions have been reported, where spermatozoa appear to have a single centriole or no centrioles at all (Fuller, 1993; Manandhar et al., 2005; Sun and Schatten, 2007; Dias et al., 2015). Perhaps a second centriolar structure exists, but electron microscopy was unable to detect it, as was the case for the *Drosophila's* second centriolar structure, the PCL (Blachon et al., 2009). The failure to recognize a second centriolar structure in most animals leaves the origin of the two zygotic centrioles and the number of centrioles in the zygote up for debate.

**Figure 8:**
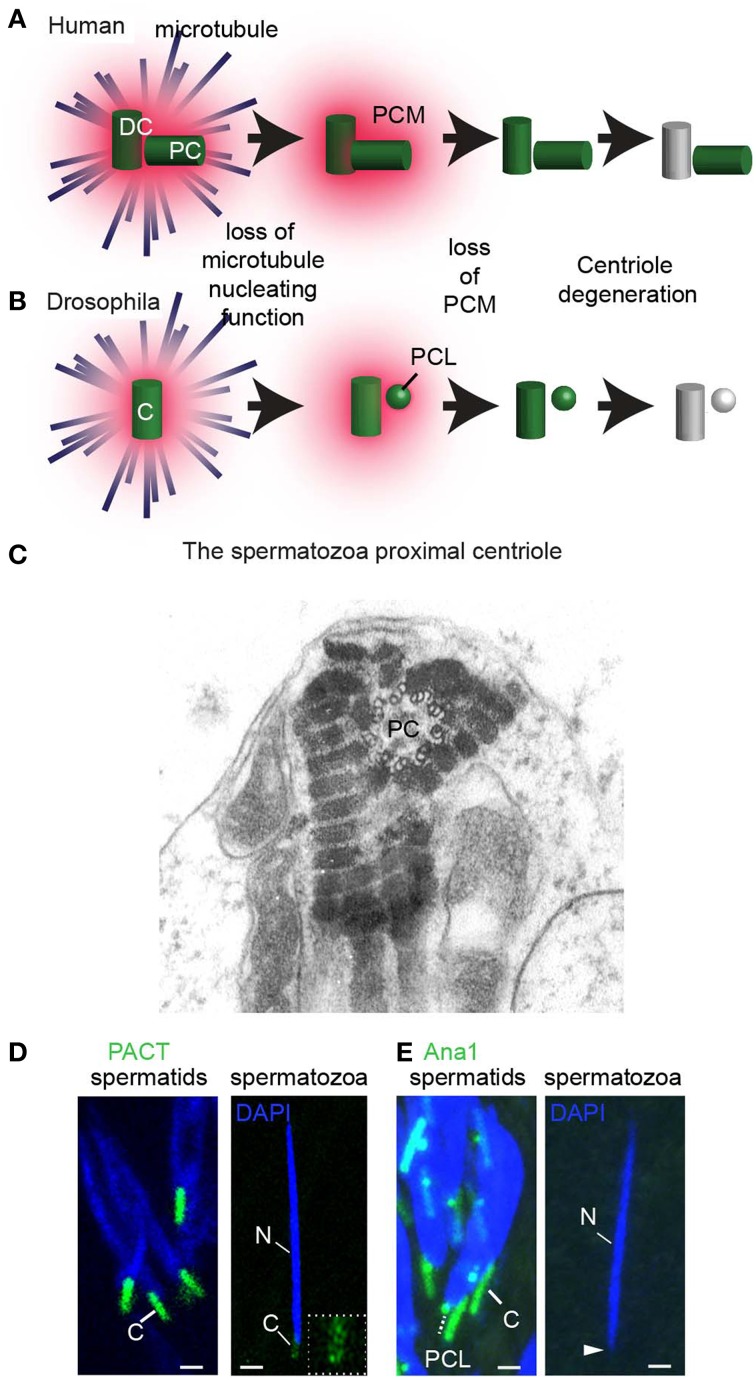
**Centrosome reduction occurs in three steps. (A)** Centrosome reduction in humans. The centrosome that has the future distal centriole (DC) and proximal centriole (PC) first loses its ability to nucleate astral microtubules. Next, it loses its PCM, and then finally, its centriolar proteins, leaving one centriole with intact microtubular structures and one degenerated. **(B)** In *Drosophila*, centrosome reduction occurs in the same three steps. Both the PCL and the centriole then undergo reduction, losing most, but not all of their comprising proteins, and the centriole degenerates. **(C)** An EM picture of the human proximal centriole (PC) in the connecting piece of spermatozoa, showing clear triplet microtubules with 9 fold symmetry. Obtained with permission from Rawe et al. (2008). **(D,E)** In *Drosophila melanogaster*, the PCL and centriole (C) undergo centrosome reduction (Blachon et al., 2014). **(D)** The centriole (C, white solid line) is intensely labeled by PACT-GFP (which is over-expressed by the strong ubiquitin promoter in intermediate spermatids), but is barely observed at the base of the sperm nucleus (see inset for magnification of this giant centriole). **(E)** The PCL (white dashed line) and centriole (C, white solid line) are observed in intermediate spermatids by Ana1-GFP. However, Ana1-GFP is not observed in spermatozoa found in the seminal vesicle (white arrowhead). Scalebar 1 μm (Blachon et al., 2014).

**Figure 9:**
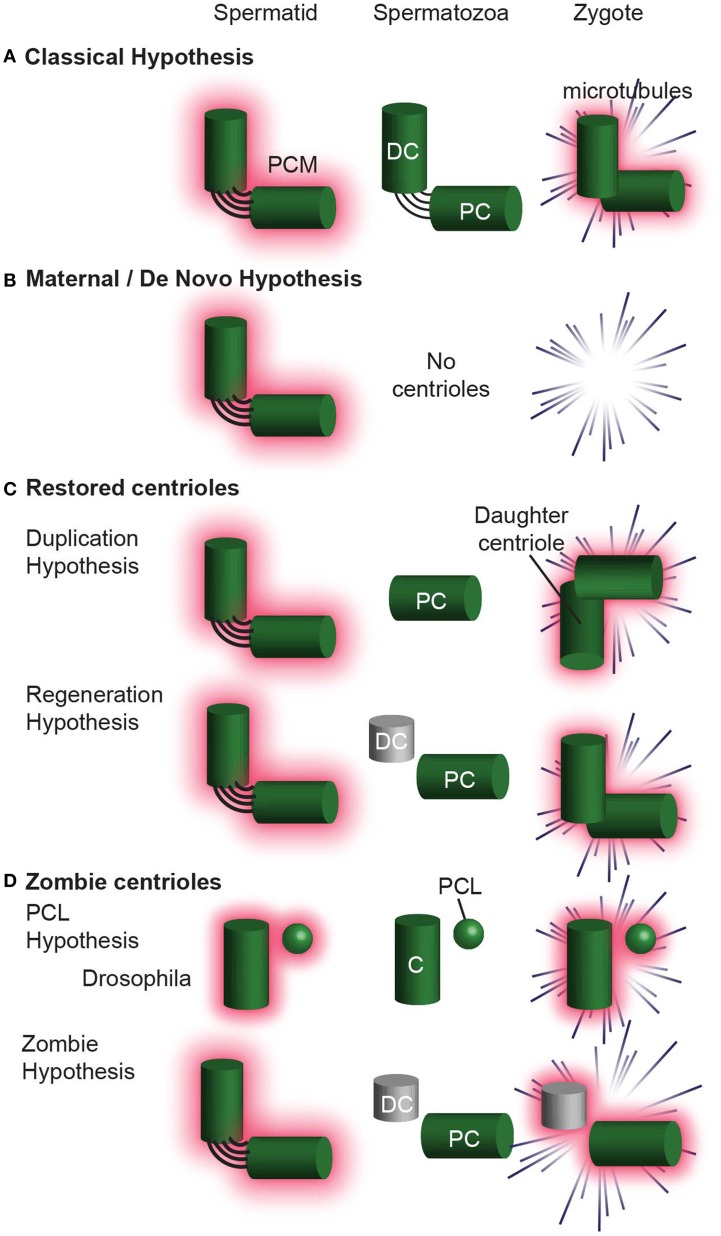
**Hypotheses for centriole Inheritance. (A)** The classical hypothesis, where, after centrosome reduction, two centrioles are inherited, and they form the zygote centrosome. **(B)** The maternal/*de novo* hypothesis, in which the spermatid has two centrioles, which are then eliminated in the spermatozoa. The zygote then undergoes division without centrioles. **(C)** Hypotheses that involve centrioles degenerating and then being restored to their original configuration upon fertilization. In the Duplication Hypothesis, one centriole is eliminated, and the second centriole is restored using the remaining centriole as a template. The Regeneration Hypothesis suggests that the distal centriole partially degenerates, but is restored upon fertilization. In this hypothesis, the centriole is only functional after restoration. **(D)** The Zombie Hypotheses state that degenerated centrioles may still be functional. For example, in mammals, the degenerated distal centriole is inherited into the zygote and functions without being restored. Similarly, the PCL is partially degenerated during spermiogenesis, but is inherited into the zygote and is functional. DC, distal centriole; PC, proximal centriole, C, centriole.

Since the centriole number, structure, and composition is distinct in the sperm of various species, several hypotheses were proposed to explain the origin of centrioles. These hypotheses can be divided into four groups: (1) The Classic Hypothesis (Figure 9A), (2) Non-Paternal Models (Figure 9B), (3) Restored Centrioles Models (Figure 9C), and (4) Paternal Precursor Models (Figure 9D). These hypotheses are not necessarily universal, as certain hypotheses have been accepted over others in specific species. Furthermore, some of these models could be combined, while others are fundamentally mutually exclusive.

#### The classic hypothesis

This hypothesis is most intuitive, stipulating that two centrioles are found in spermatozoa and that these two centrioles enter the zygote and function there. These centrioles are intact; centrosome reduction eliminates only their PCM. This model of inheritance was suggested in *C. elegans*, sea urchin, and *Xenopus Laevis*.

#### Non-paternal centriole

These models attempt to explain the observation that in some rodents (e.g., mice and rat) and some insects, both the sperm and the zygote lack recognizable centrioles, yet centrioles are observed in the embryo. For example, centrioles appear when the embryo reaches the 32 or 64 cell stage of these rodents (Schatten et al., 1986; Gueth-Hallonet et al., 1993). There are two mutually exclusive models:

*The Maternal Precursor Hypothesis:* This hypothesis postulates that the ovum contains a currently unrecognizable centriolar precursor that somehow gives rise to the embryo's centrioles (Calarco, 2000).

*The de novo Hypotheses:* This hypothesis postulates that the centrioles form *de novo* in the embryo (Courtois et al., 2012).

#### Restored centrioles

These models attempt to explain the observation that in non-rodent mammals and other vertebrates, two or more centrioles are observed in the zygote even though spermatozoa appear to have only one intact centriole. There are two mutually exclusive models:

*The Duplication Hypothesis* postulates that a single functional centriole is inherited from the sperm and is duplicated shortly after fertilization, in the absence of DNA duplication (Sutovsky and Schatten, 2000). In non-rodent mammals, the single functional centriole in the zygote is known to be the proximal centriole. The second centriole in the zygote is presumed to be the proximal centriole's daughter centriole. These two centrioles are thought to duplicate in the zygote, parallel to DNA duplication, resulting in four centrioles during the first zygotic mitosis. This model has a potentially fatal flaw, as only three centrioles have ever been observed using serial section electron microscopy during the zygotic mitosis (Sathananthan et al., 1996; Crozet et al., 2000). This model was thought to occur in Drosophila too (Riparbelli et al., 1997), however this model was disproved (Blachon et al., 2014).

*The Regeneration Hypothesis* attempts to explain the observation that in non-rodent mammals two centrioles are found in early spermatids, while in the spermatozoa one is intact, and the second is degenerated. This hypothesis proposes that after fertilization, the degenerated centriole regenerates to form a second centriole (Schatten and Sun, 2009). Like the duplication hypothesis, this model assumes that four centrioles exist during the first zygotic mitosis, although only three centrioles have ever been observed during the zygotic mitosis (Sathananthan et al., 1996; Crozet et al., 2000).

#### Paternal precursor

These models are based on recent data and revisiting preexisting literatures. Recent data suggests that the PCL of *D. melanogaster*, which does not have microtubules, is the second sperm and zygotic centriole (Blachon et al., 2009, 2014). Revisiting of preexisting literature suggests that the centriole of insect spermatozoa is degenerated, as it completely lacks or has a disorganized array of microtubules. This degenerated insect centriole is similar to the degenerated distal centriole in non-rodent vertebrates. Therefore, we now propose two new hypotheses that apply to insects as well as to certain mammals. These hypotheses are mutually exclusive with the *Duplication Hypothesis*, *de novo Hypotheses, Regeneration Hypothesis*, and *The Maternal Precursor Hypothesis*, but are not mutually exclusive to each other.

*The PCL Hypothesis* attempts to explain the observation that only one centriole is present in insects throughout spermiogenesis. This hypothesis proposes that the 2nd centriolar structure is the proximal centriole-like (PCL) (Blachon et al., 2009). In the zygote, the PCL acts as the second centriolar structure, despite the absence of centriolar microtubules (Blachon et al., 2014). The PCL never was a typical centriole and is similar to an arrested early-intermediate in centriole formation. The PCL hypothesis assumes that the PCL does not need to regenerate its 9-fold symmetric microtubules in order to function (i.e., recruit PCM, form asters, and “duplicate” to form a new centriole). Evidence for this model is present in Drosophila and it is possible that a PCL-like structure is present in other insects, non-rodent mammals, and rodents however no evidence for that is currently available.

*The Zombie Hypothesis* is a new hypothesis that claims that in many animals, after forming a typical centriole(s), one or more centrioles undergo dramatic centrosome reduction, resulting in their structural and compositional degeneration. Yet, these degenerated centrioles are functional (i.e., recruit PCM, form asters, and “duplicate” to form a new centriole), and do not need to regenerate their 9-fold symmetric microtubules to do so. Because these centrioles are both degenerated (“dead”) and functional (“live”), we named them zombie centrioles.

The PCL and zombie hypotheses, while conceptually similar, are different in that the PCL never fully develops into a typical centriole whereas the zombie centriole developed into a typical centriole, and then degraded, leaving a remnant. Some evidence suggests that this model applies to the single centriole of insect sperm. In addition, while no direct evidence currently exists, zombie centrioles maybe present in non–rodent mammals. A somewhat similar idea suggested that in non–rodent mammals the unrestored, degenerated centriole duplicates to give rise to a centriole; however, in this model, the degenerated centriole is not able to form an aster (Schatten and Sun, 2009).

### What evidence exists to support the classic hypothesis of centriole inheritance?

In the classic hypothesis, the spermatozoon provides two centrioles to the zygote. This was shown convincingly in *C. elegans*, the mollusk *Crassostrea virginica* (American oyster) and sea urchin. In *C. elegans*, electron microscopy showed that immediately after fertilization, the zygote has two centrioles, made of singlet microtubules with 9-fold symmetry (Figure 3 in Pelletier et al., 2006). These findings are consistent with light microscopy studies showing centriolar proteins Sas-6-GFP and Sas-4-GFP from the sperm are maintained in the zygote centriole. This suggests that *C. elegans* centrioles are intact. Likewise, in the mollusk *C. virginica*, electron microscopy found that ejaculated spermatozoa have intact proximal and distal centrioles (Figures 5, 8 in Daniels et al., 1971).

Consistent with the classic hypothesis, in sea urchin zygotes, a pair of centrioles is found at each end of the spindle pole during mitosis, with microtubules in nine-fold symmetry (Greenfield Sluder, unpublished data) (Figures 2, 3 in Sluder and Rieder, 1985a). Another electron microscopy study claims that the proximal centriole (a.k.a. mitochondrial centrosome in Paweletz et al., 1987) is degenerated while the distal centriole is intact (a.k.a. sperm head centrosome in Paweletz et al., 1987). However, since the ultrastructure preservation appears to have been imperfect in the latter paper, the classic hypothesis may still apply to sea urchin.

In addition, there is evidence supporting the classic model for *X. Laevis*. In *X. Laevis*, electron microscopy of testicular spermatozoa found intact proximal and distal centrioles (Figures 8, 11 in Bernardini et al., 1986; Figure 6 in Felix et al., 1994). However, whether these centrioles are reduced or degenerated in post-testicular stages, as they are in mice, has not been investigated.

Interestingly, two intact centrioles are observed in the spermatozoa of two phylogenetically distinct organisms, *C. elegans* and *Cancer crab*, which both lack flagella (Figure 28 in Langreth, 1969). Since in many species with flagellated spermatozoa centrioles are degenerated, the above observation suggests that the presence of flagella is connected to the presence of abnormities in spermatozoa centrioles. It will be interesting to study whether other non-flagellated spermatozoa also have intact centrioles.

### What evidence exists that supports the non-paternal centriole hypothesis?

In rodents (mouse and rat), it was suggested that the sperm does not provide any centriolar structures at fertilization. Instead, centrioles appear at the 32/64-cell stage (Courtois et al., 2012). Several lines of investigation led to this proposal: (1) Electron microscopy studies have not been able to identify proximal nor distal centrioles in the ejaculated spermatozoa (Figure 7 in Woolley and Fawcett, 1973). (2) Electron microscopy studies have not been able to identify centrioles in the zygote (Zamboni et al., 1972) (3) Immunofluorescence and live imaging studies in the zygote did not identify a dominant microtubule aster associated with the fertilizing sperm head (Schatten et al., 1985, 1986; Courtois et al., 2012). Instead, immediately post-fertilization, PCM starts to aggregate randomly in the cytoplasm and forms mini-asters that are recruited to the pronuclei membrane, where they are organized into a barrel-shaped spindle (Courtois et al., 2012). These cytoplasmic mini asters of the mouse zygote resemble the mini asters observed in parthenogenetic embryos of non-rodent mammals that divide in the absence of paternal centrioles (Morito et al., 2005; Terada et al., 2009). This pattern of aster formation suggests that the paternal centrioles that are normally responsible for PCM recruitment and concentration are absent or are unable to form a dominant microtubule aster.

For the maternal/*de novo* hypotheses to be valid, there needs to be non-centriolar mechanisms that perform the essential functions that are carried out by the centriole in other models. This includes: (1) pronuclear migration and congregation; (2) organization of the spindle pole; and (3) precise regulation of centriole number. In response to this first function, it was proposed that pronuclear migration is mediated by actin filaments in mice (Maro et al., 1984). Organization of the spindle pole can be achieved in the absence of centrioles in many cell types (Hinchcliffe et al., 2001; Basto et al., 2006), including in the fertilized ovum during female meiotic division (Szollosi et al., 1972; Sathananthan et al., 2006; Luksza et al., 2013). How regulation of centriole number takes place in the absence of a preexisting centriole or centriolar structure is not clear.

Like mice and rats, stick insects of the *Bacillus* genus are thought to have no centrioles in spermatozoon or in zygotes. Two lines of evidence are available. First, electron microscopy studies do not find centrioles in *Bacillus* spermatozoon, suggesting that the spermatid single centriole is modified or completely degenerated (Baccetti et al., 1973). Second, in *Bacillus* zygotes, no sperm asters are associated with the fertilizing sperm head, suggesting that the spermatozoa do not contribute a functioning centriole (Marescalchi et al., 2002).

### What evidence exists to support the restored hypothesis?

There is no evidence that the degenerated centrioles in sperm regain microtubules with 9-fold symmetry in the zygote, as proposed by the regeneration model. Instead, it was reported in serial section analyses of both sheep and human zygotes that only three centrioles were identified (Sathananthan et al., 1996; Crozet et al., 2000). In both the sheep and human zygotes during cell division, one spindle pole had two electron dense centrioles, and the other pole had only one centriole. Some of the zygotic centrioles in humans have microtubules, but this may be restricted to the proximal centriole in the zygote inherited from the sperm. One way to explain these findings is that the pole with two centrioles has the proximal centriole and its daughter centriole. The other pole should also have two centrioles according to the *Regeneration* and *Duplication* model, but there is only one centriole.

The proximal centriole was proposed to be the only centriole provided by the human sperm to the zygote, and that it is a precursor to the four centrioles in the zygote during its division (Sathananthan et al., 1996). According to the duplication model, the proximal centriole duplicates first during the two-pronuclear zygote (2PN) stage. These centrioles form one of the spindle poles. Then they duplicate, and one pair relocates to the other pole. However, there is no direct evidence for the presence of four centrioles during zygote cell division, or for the relocation of the pair of centrioles during cell division. This lack of evidence is consistent with alternative idea that centrioles are present but are atypical (PCL and zombie centrioles).

### What evidence exists to support the PCL hypothesis?

**Figure 10:**
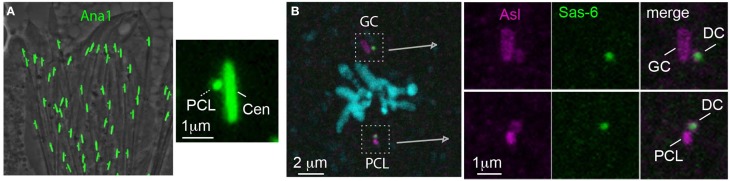
**The PCL is a centriole precursor that forms in the spermatid and becomes one of the zygote's spindle poles. (A)** Ana1-GFP labels the PCL that forms near the Ana-1 labeled giant centriole (GC) in *Drosophila* spermatids (Blachon et al., 2009). **(B)** A metaphase zygote formed from an ovum expressing Sas-6-GFP has both a PCL and GC centrosome (which are labeled by antibody against the PCM marker Asl). The PCL and GC centrosomes each have their own daughter centrioles (DC) (which are labeled by the maternal Sas-6- GFP) (Blachon et al., 2014).

A paternal precursor model was proposed initially by Crozet et al. (2000) as one of the mechanisms that can account for their observation that sheep zygotes during cell division have three centrioles instead of the expected presence of four centrioles. However, the first evidence for the existence of paternal precursors came from the study of centrioles in *D. melanogaster* (Blachon et al., 2009). In *D. melanogaster*, the spermatids have one centriole that resembles the distal centriole because it nucleates the flagellum (Tates, 1971; Tokuyasu, 1975; Carvalho-Santos et al., 2012). An additional atypical centriole was discovered as a result of studying the localization of the centriolar protein Ana1 (the ortholog of human Cep295, also known as KIAA1731) in spermatids. It was found that spermatids have two Ana1 foci; one elongated, as expected from the giant centriole, and one unexpected small focus (Figure 10A). During spermiogenesis, the small Ana1 focus was found first at the proximal end of the centriole and was therefore named the Proximal Centriole-Like (PCL) (Blachon et al., 2009). Further studies found that the centriolar proteins Plk4, Sas-6, Ana2, Sas-4, and Bld10 and the PCM proteins Asl, Cnn, and γ-tubulin mark the PCL (Blachon et al., 2009; Stevens et al., 2010). In addition, PCL formation is dependent upon the same proteins that are involved in the initiation of centriole formation, such as: Plk4, Sas-6, and Asl (Blachon et al., 2009). This suggests that the PCL is related in its origin to a centriole. However, unlike centrioles, the PCL does not have microtubules, nor does it require Sas-4 for its formation, which is essential for centriole microtubule formation (Blachon et al., 2009). This suggested that the PCL is a centriole precursor that lacks microtubules.

After the discovery of the PCL in spermatids, the question became: is the PCL inherited by the zygote? This question is critical, because in late spermiogenesis spermatids lose most of their cytoplasmic content in the processes known as individualization and spermiation (Noguchi and Miller, 2003; Xiao and Yang, 2007). In addition, like in the centriole, centrosomal proteins do not mark the PCL in spermatozoa because of centrosome reduction (Blachon et al., 2014) (Figure 10B). Ultimately, it was shown that the PCL is delivered from the sperm to the zygote in two independent experiments that excluded the possibility that the 2nd centriole of the zygote is of maternal or zygotic origin. It was found that in the zygote, like the centriole, the PCL recruits PCM, forms an aster, serves as a scaffold to form one daughter centriole, and is found in one of the zygotic spindles during division (Figure 10B) (Blachon et al., 2014). The PCL was only recently discovered, and additional directed studies are necessary to understand how it forms and functions, and whether a PCL is present in other animals.

#### Naegleria pringsheimi novel centriolar precursor structure

Most interestingly, in the single cell amoeba *Naegleria pringsheimi*, a recent study has identified precursors for centrioles, which explains how centrioles are inherited in this system that was thought to be governed by *de novo* centriole formation (Lee et al., 2014). *Naegleria* have two body forms: an amoeba and flagellate. The amoeba form undergoes mitosis and the flagellate is a non-dividing differentiated form. The amoeba lacks centrioles, but the two centrioles appear during differentiation to the flagellate. These two centrioles originate during differentiation from transient precursors known as GPM complexes, which contain γ-tubulin, pericentrin-like protein, and myosin II (Kim et al., 2005). Importantly, the GPM complex originates from a self-replicating precursor that is present in the amoeba and was named GPMp (Lee et al., 2014). The GPMp contains a transacylase that marks it specifically and disappears once the centriole forms. This finding demonstrates that even in a system where centrioles appear to assemble *de novo*, the reality is that they are assembled from a precursor with a distinct composition. Similar precursors may be present in mice and rats and could explain the origin of their centrioles.

### What evidence exists to support the zombie hypothesis?

Classic electron microscopy studies concluded that in many insects, the spermatid centrioles with 9 fold symmetry of microtubules disappear in mature sperm (Phillips, 1970). This is supported by observations from more recent electron microscopy studies of Drosophila's spermatozoa (Blachon et al., 2009; Yasuno and Yamamoto, 2014). In these observations, the microtubules of the centriole appear collapsed into a tight bundle at the proximal end. Taking advantage of the long centriole found in some insect sperm, a light microscopy study unequivocally demonstrated that the giant centriole, despite its collapsed structure, functions as the zygotic centriole (Friedlander, 1980). Similar observations were made recently in *D. melanogaster* using fluorescent microscopy (Blachon et al., 2014). These seemingly contradictory findings can be reconciled by the idea that the centrioles of insect spermatozoa are extremely modified/degenerated; yet can function as centrioles after fertilization (i.e., they are zombie centrioles). Similar arguments can be made for the vertebrate's distal centriole.

It appears that like the PCL, the atypical insect zombie centriole can direct the formation of a daughter centriole. If a zombie centriole (the degenerated distal centriole) is present in mammals, it would fit the observation of three centrioles in the human and sheep metaphase zygote (Sathananthan et al., 1996; Crozet et al., 2000). However, in mice, where centrioles are not observed up to the 32/64-cell stage (blastocyst), zombie centrioles are expected to duplicate for several cell cycles prior to forming centrioles. Presumably, these centriole structures are initially ineffective in recruiting the PCM, and they develop the ability to recruit PCM later. This postulated mechanism would fit the observation that in mouse early embryos, the transition from meiotic spindle assembly, which lacks centrioles, to mitotic spindle, which is centriolar, is gradual (Courtois et al., 2012).

#### Mice may contribute a degenerated centriole that functions in the zygote to duplicate centriole

Although, there is a general belief that mice spermatozoa do not contribute centrioles to the zygote, several recent studies suggest that a paternal centriolar structure is contributed to the zygote. First, injection of mouse spermatozoa into cat ovum results in the formation of asters at the base of the sperm nucleus immediately after fertilization, suggesting that the sperm does provide a microtubule organization center (Figure 3B in Jin et al., 2012). This fertilization results in formation of bipolar spindles and successful zygote division. This interesting study suggests that the mice ovum is different from that of cat or other mammalian ova in the way it forms astral microtubules, but not in the presence of a centriolar structure. Second, a study of the protein Speriolin, a sperm centriole protein, also suggests that a centriolar structure may be present in mouse spermatozoa (Figures 3, 7 in Goto et al., 2010). Third, spindle formation in the mouse zygote requires Plk4 (Coelho et al., 2013). Since Plk4 is the master regulator of centriole formation, this observation suggests that a centriolar structure is present.

Similar to other mammals, electron microscopic analysis in the rodents: chinchilla, Guinea pig, Chinese hamster, and ground squirrel, suggest that the proximal centriole is retained but the distal centriole is degenerated in the spermatozoa (Fawcett, 1965; Fawcett and Phillips, 1969 #3867). Therefore, it appears that the dramatic degeneration of both the proximal and distal centriole is limited even in rodents.

### What is the significance of the paternal precursor centriole hypothesis?

One significant aspect of the paternal precursor centriole hypothesis is that it proposes a universal mechanism of centriole inheritance among animals. It might be that two centriolar structures are inherited from the spermatozoa in all animals; however these structures are not always typical centrioles. The two structures may be two intact centrioles, two degenerated centrioles, two PCLs, two GPMs, or any combination of these.

Another significant aspect of the paternal precursor centriole hypothesis is that it may provide insight into how the zygote centriole duplicates and functions. One idea to explain centriole duplication is that the preexisting centriole (or centriolar structure) functions as a template for a new centriole. This idea of “template” fell out of favor because centrioles can form *de novo* (Rodrigues-Martins et al., 2007b). However, recently, a new idea that attempts to explain centriole duplication is that the proximal lumen of the preexisting centriole functions as a counting device to restrict centriole number (Fong et al., 2014). Alternatively, it could be that a particular triplet microtubule of the 9 triplet microtubules of the centriole is unique and determines the site of new centriole formation (O'Toole and Dutcher, 2014). If the degenerated centriole does not have microtubules with 9-fold symmetry, then there is no proximal lumen. If the PCL does not have microtubules to determine the site of new centriole formation, then we could infer that the microtubules do not determine the new centriole nucleation site. Therefore, the ability of PCL and degenerated centriole to duplicate provides useful insight to the mechanisms of centriole duplication.

The paternal precursor centriole hypothesis could shed light on the functional significance of PCM organization. The PCM and other centriole-associated structures have pseudo 9-fold symmetry that is thought to be a reflection of the microtubule 9-fold symmetry (Lau et al., 2012; Mennella et al., 2012). If the PCL or the degenerated centriole does not have microtubules with 9-fold symmetry, then their PCM in the zygote may also lack pseudo 9-fold symmetry. Therefore, PCM 9-fold symmetry would not be essential for the centrosome to function as a microtubule-organization center.

## Summary

The zygote centrioles are a key subcellular organelle for fertilization as well as for animal development and physiology. In the zygote, the centrioles form centrosomes that mediate the migration of the female pronucleus and cell division. In addition, the zygote centrioles duplicate to form essentially all of the animal centrioles, which are essential for the cilia present in most of our cells. Defects in sperm centrioles, which affect their function in the zygote, are expected to result in male infertility; however, very little is known about this type of infertility. Also, we do not yet fully understand the structural and molecular mechanisms underlying the formation, modification, and maintenance of the various centriolar structures (i.e., PCL and degenerated centrioles) in the sperm and zygote. Therefore, directed studies are needed to precisely identify the centriole proteins and organization in the spermatozoa and zygote. Beyond gaining an essential understanding of fertilization, these studies will shed light on other basic questions and mechanisms in cell and developmental biology, such as centriole function, centriole duplication, and PCM formation.

### Conflict of interest statement

The authors declare that the research was conducted in the absence of any commercial or financial relationships that could be construed as a potential conflict of interest.

## Abbreviations

PCL: proximal centriole-like
PCM: pericentriolar material
GC: giant centriole.
